# 3D Rapid Prototyping Heart Model Validation for Teaching and Training - A Pilot Project in a Teaching Institution

**DOI:** 10.21470/1678-9741-2020-0433

**Published:** 2021

**Authors:** Sivakumar Krishnasamy, Raja Amin Raja Mokhtar, Ramesh Singh, Sivakumar Sivallingam, Yang Faridah Abdul Aziz, Vickneswaran Mathaneswaran

**Affiliations:** 1 Division of Cardiothoracic Surgery, Faculty of Medicine, University Malaya, Kuala Lumpur, Malaysia.; 2 Division of Cardiology, University Malaya Medical Centre, Kuala Lumpur, Malaysia.; 3 Department of Cardiothoracic Surgery, National Heart Institute, Kuala Lumpur, Malaysia.; 4 Department of Biomedical Imaging, Faculty of Medicine, University Malaya, Kuala Lumpur, Malaysia.; 5 Division of Neurosurgery, Faculty of Medicine, University Malaya, Kuala Lumpur, Malaysia.

**Keywords:** Imaging, Three-Dimensional, Models, Anatomic, Printing, Three-Dimensional, Coronary Sinus, Coronary Vessels, Heart

## Abstract

**Introduction:**

Rapid prototyping is a process by which three-dimensional (3D) computerized surface models are converted into physical models. In this study, a 3D heart bio model was created using the rapid prototyping method and the accuracy of this heart model was assessed by clinicians.

**Methods:**

The two-dimensional images of normal heart from gated computed tomography scan datasets were used to create a 3D model of the heart. The slices were then processed using the software BioModroid and printed with the 3D printer. The evaluation of the model was performed by a questionnaire answered by four cardiothoracic surgeons, 12 cardiologists, five radiologists, and nine surgical registrars.

**Results:**

Eighty-six percent of the anatomy structures showed in this model scored 100% accuracy. Structures such as circumflex branch of left coronary artery, great cardiac vein, papillary muscle, and coronary sinus were each rated 77%, 70%, 70%, and 57% accurate. Among 30 clinicians, a total of 93% rated the model accuracy as good and above; 64% of the clinicians evaluated this model as an excellent teaching tool for anatomy class. As a visual aid for surgery or interventional procedures, the model was rated excellent (40%), good (50%), average (23%), and poor (3%); 70% of the clinicians scored the model as above average for training purpose. Overall, this 3D rapid prototyping cardiac model was rated as excellent (33%), good (50%), and average (17%).

**Conclusion:**

This 3D rapid prototyping heart model will be a valuable source of anatomical education and cardiac interventional management.

**Table:** 

Abbreviations, acronyms & symbols	
2D	= Two-dimensional
3D	= Three-dimensional
CT	= Computed tomography
DICOM	= Digital Image Communication in Medicine
ECG	= Electrocardiogram
H	= Hounsfield
MRI	= Magnetic resonance imaging
RP	= Rapid prototyping
RPT	= Rapid prototyping techniques
WH	= Wave height
WL	= Wavelength

## INTRODUCTION

Rapid prototyping (RP) is a process by which three-dimensional (3D) computerized surface models are converted into physical models. This print technology was developed in the 1980s and for some time it was used only in industry. It was originally developed to design components for numerous products, including automobiles, aircraft, and computers. With the unique capabilities afforded by modern imaging modalities, such as multi-detector computed tomography (CT), magnetic resonance imaging (MRI), and 3D echocardiography^[1]^, the use of RP has been extended to medical applications.

The introduction and development of new computer techniques and softwares have enabled the visualization and creation of virtual and real anatomical models. 3D modeling is a development in the medical field, which has given accurate details on the physical structure of human organs. These models give students the opportunity to learn anatomy in detail and practice medical procedures repeatedly as many times as necessary. RP techniques (RPT) offer new possibilities both in the field of medicine and in medical education.

Historically, preserved cadaver hearts obtained at autopsy were used to teach anatomy. 3D models of the specimens were created using paraffin wax or silicone casting. These methods, however, were time-consuming, could only be performed on postmortem specimens, and involved a process necessitating the destruction of the casted heart^[2,3]^. The autopsy specimens may or may not have anatomy similar to that of the patient. Furthermore, cardiac chambers in autopsy specimens are either collapsed or frozen at the end of diastole or the end of systole depending on the fixative. The issue of cost, in addition to the short duration of usage and availability, makes it even more difficult and inefficient to acquire cadavers with specific pathology. As such, a 3D prototype heart model will be of higher integrity and inject increased realism in anatomy classes.

3D modeling has seen a widespread successful usage in surgical fields such as maxillofacial surgery for mandibular reconstruction^[4,5]^ and neurosurgery for cranial implants^[6-8]^ because of the unique nature of each individual's skull structure.

Proper understanding of anatomical relations is crucial in cardiovascular surgery. Abnormalities occurrences in the cardiovascular system such as congenital heart disease, aortic stenosis, and pulmonary atresia defect drive the search for an assistance and training module for current and future cardiologists or cardiac surgeons. Introduction to 3D-based imaging technologies shows promising future and benefits in improving the quality of case assessment, diagnosis, and surgical procedure^[9-11]^. 3D imaging solves the misinterpretation in two-dimensional (2D) images. By 3D image reconstruction, the abnormalities of the cardiac morphology can be viewed at any angle, observed, and identified^[12]^.

3D prototyping of the replicas certainly allows direct visual access to the physical structures and, more importantly, direct physical manipulation, such as practice surgery on the replica of the structure to be operated. Even more exciting, they have the potential to be used in an interactive training environment for both existing and novel catheter-based procedure. Surgeons and interventionists can use patient-specific physical models pre-procedurally to appreciate potential procedural challenges, assess the likelihood of success or failure, and to select appropriate equipment and devices for use^[10]^.

At the visionary end of this spectrum is the idea of investigating biological materials (stem cells, etc.), in the hope of 3D printing cells, valves, vessels, or even organs, *e.g*., the entire heart. There would no longer be any organ shortage and patients with terminal heart failure would get their new hearts from their own cells without the need for postoperative medications. Bypass materials would be made of human cells. Even though this may still be science fiction today, just dare to think about the new treatments in fields for which there are no treatment options yet available. In China, mini-kidneys are already produced^[13]^. However, there is no doubt this cutting-edge technology could also be used for ethically questionable purposes, *e.g*., to print embryos, weapons, etc.

In conclusion, 3D prototype rapid printing of a normal heart is only the first in a series of projects to produce diseased heart models. RP of heart models provides an opportunity for individuals to hold and visualize the human heart (normal or pathological), as well as to practice medical procedures. Though discrepancies still remains or exist^[14]^, the 3D models are more beneficial in medical field.

The objectives of this study were:

Primary Outcomes

1. To create a 3D RP cardiac model.
2. To verify the anatomical accuracy of the model among clinicians.

Secondary Outcome

1. To evaluate the usefulness of this model in the future medical field.

## METHODS

**Fig. 1.1:**
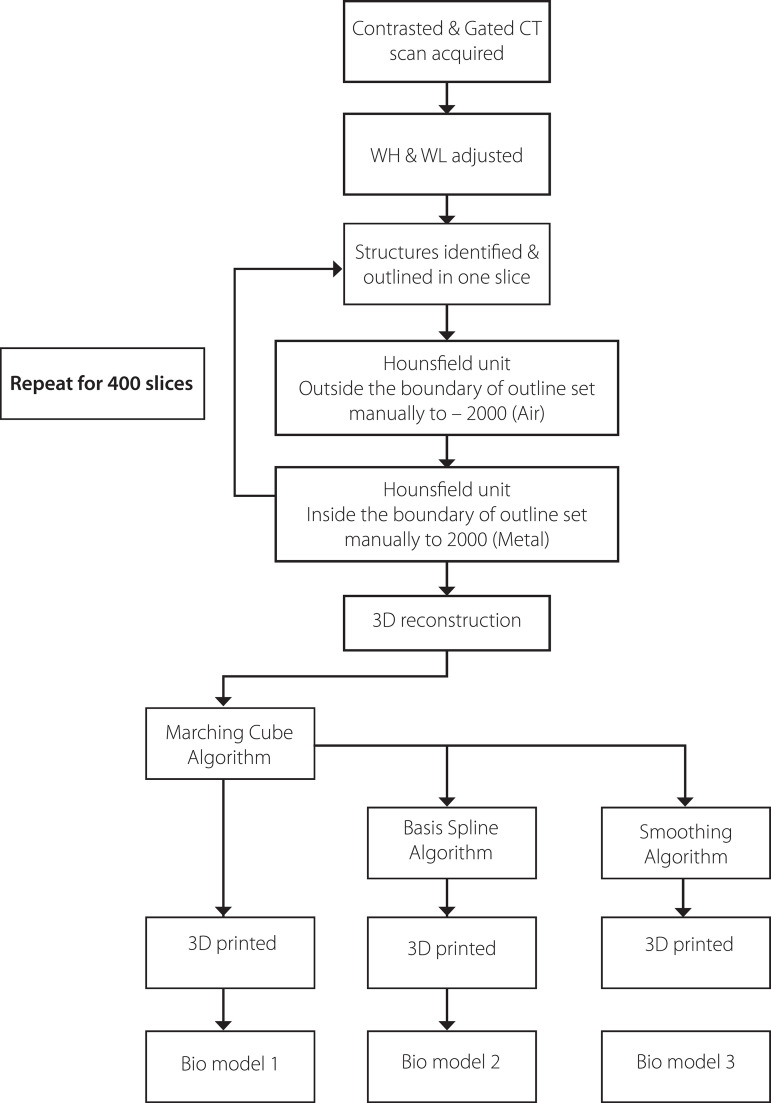
Process flow of the study. 3D=three-dimensional; CT=computed tomography; WH=wave height; WL=wavelength

In this study, 400 electrocardiogram (ECG)-gated CT scans with a thickness of 600 µm each and gantry angle of 0º were obtained using a fine slice method. A flow chart of the process can be referred to in Figure 1.1. These scans were in Digital Image Communication in Medicine (DICOM) format and were then imported into BioModroid, which is a biomodelling software.

**Fig. 1.2:**
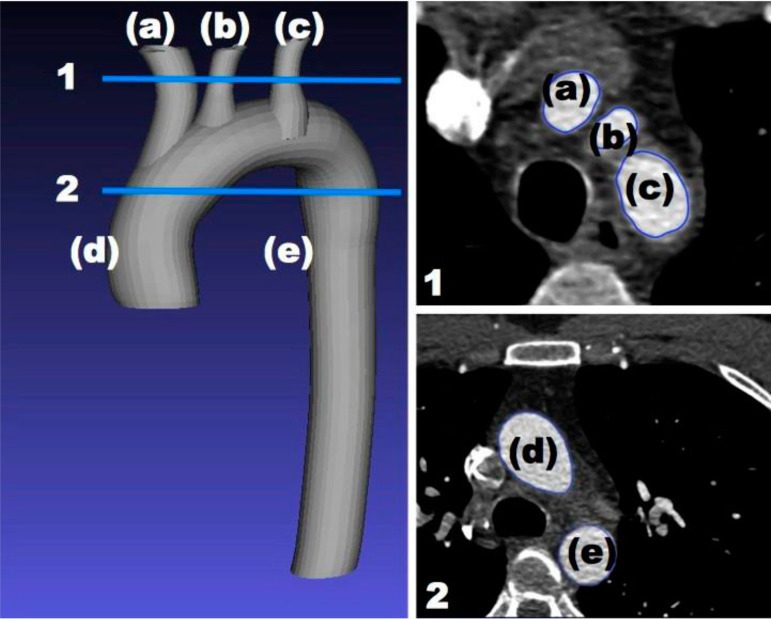
Substructures of the heart model in this study shown in relation to two slices out of the 400 slices of computed tomography scans. The structures are the ascending aorta (d), descending aorta (e), subclavian artery (a), left common carotid artery (b), and brachiocephalic artery (c). The two slices were captured on a two-dimensional plane marked with horizontal lines (1&2).

The outline of the cardiac structures of interest were done first prior to reconstructing the 2D images into a 3D model. The substructures identified for this study were the ascending and descending aorta, subclavian artery, left common carotid artery, and brachiocephalic artery (Figure 1.2).

**Fig. 1.3:**
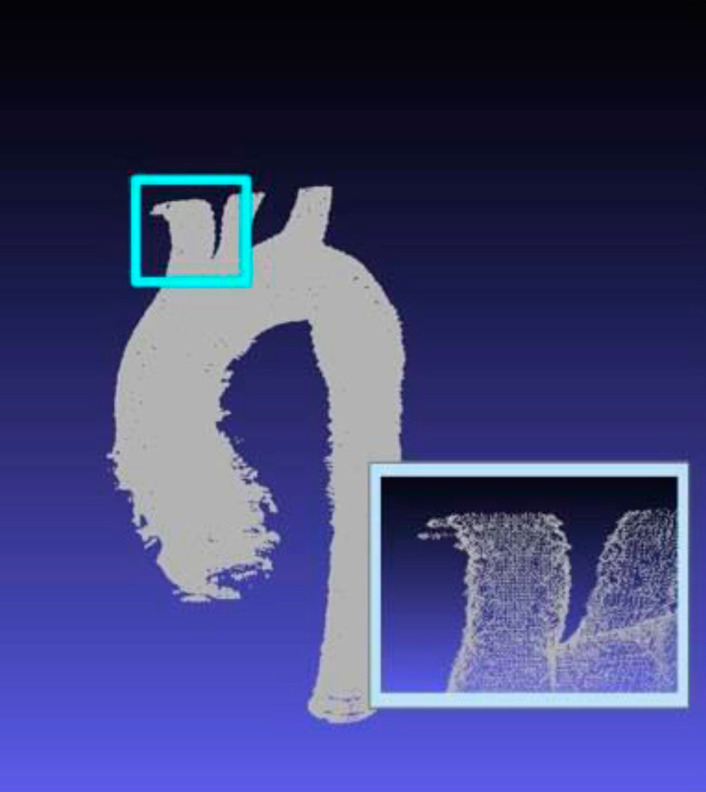
The multiple dots that make up the dataset. The brachiocephalic artery and portions of the left common carotid artery are magnified in the inset.

In the first step of the image processing, major blood vessels were outlined. The resulting data were in multiple 3D points as depicted in Figure 1.3. The multiple points are visible in the inset. Any inward curves and random points were accounted for and corrected. The Hounsfield (H) units of the data points outside the outlines were then adjusted to -2000 H (the value of air) and the points within the outline were set to 2000 (H value of metal). This process was repeated for each of the 400 slices. Having completed this initial stage, the images were stacked in the volume rendering stage to create a 3D model of these structures. To ensure this was done as accurately as possible, the scans must be overlapped with careful precision.

Then three 3D construction algorithms - the basis spline, the Marching Cubes, and the smothering on Marching Cubes - were applied on the model to be compared to identify which algorithm was best suited for processing the cardiac data. RP apparatus - 3D systems powder-based ZCorp Z450 (RPA1) and Stratasys multi-material Connex500 (RPA2) - were utilized to produce a 3D print in physical form for each of the algorithms (resulting in three bio models) as output indicator for validating and testing the outcome of the algorithms.

Once suitable algorithms were created, the left-sided heart chambers, followed by right-sided heart chambers, pulmonary vessels, heart muscles, coronary vessels, and valves, were outlined. The drawings were verified by cardiothoracic and radiology consultant before printing.

The model was printed using a 3D printer with innermost structure (chambers) made of rubber and outermost structure made of plastic materials. Wax was used to represent the heart muscles. A RP apparatus (multi-material, ultraviolet-cured Stratasys) was utilized to generate a 3D physical bio model of the myocardium. In order to best mimic the elasticity properties of the myocardium, a similar synthetic rubber-like material was used (Tango Plus). Then, a 3D physical cardiac bio model was postprocessed in order to preserve the rubber layer and remove the support material in certain unwanted areas.

Once the 3D RP heart model was printed, the model was evaluated using questionnaires that were given to four cardiothoracic surgeons, 12 cardiologists, five radiologists, and nine surgical registrars.

## RESULTS

The printed model of the normal heart is depicted in Figure 2.1 till Figure 2.7. It has been sliced so that the inner structures were able to be visualized (Figure 2.7). The produced cardiac bio model can depict various parts of the heart model, including aorta and supporting tissues. In addition, the layer of myocardium with a different colour and texture is clearly seen and can be easily identified.

**Fig. 2:**
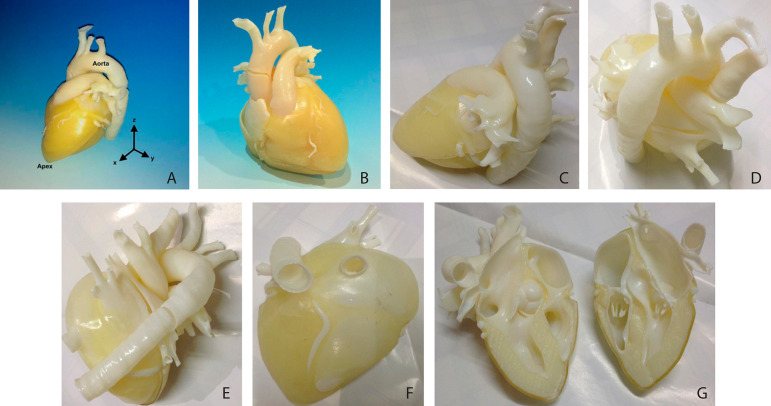
A) – Three-dimensional printed normal heart bio model. The myocardium (yellow area) is clearly visible in the model (left anterior oblique 60˚ view). B) - Posterior anterior view of heart model. C) - Left lateral view of the heart model. D) - Craniocaudal (superior) view of the heart model. E) - Posterior view of the heart model. F) - Inferior (caudal) view of the heart model. G) - Cross-sectional view of the heart model.

**Fig. 3.1:**
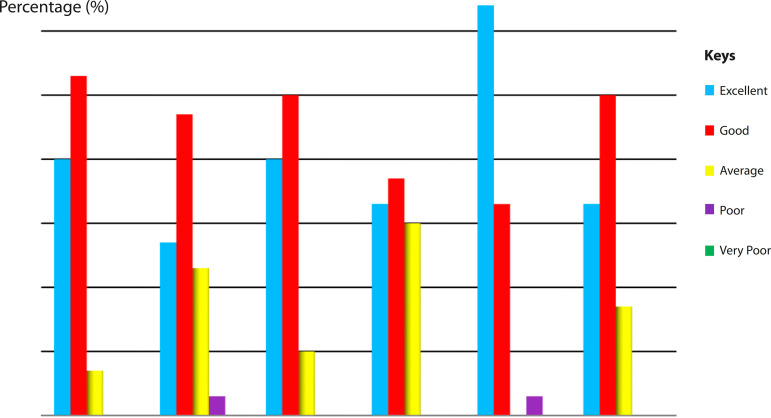
Scoring of the heart model on accuracy and usefulness in the future (n=30).

Figure 3.1 shows scoring of the heart model on accuracy and usefulness in the future. Sixty-four percent of the clinicians scored the model as excellent for anatomical teaching class, whereas only 27% of the clinicians scored the model as excellent for treatment planning. As for training purpose, nine clinicians rated the model as poor.

**Fig. 3.2:**
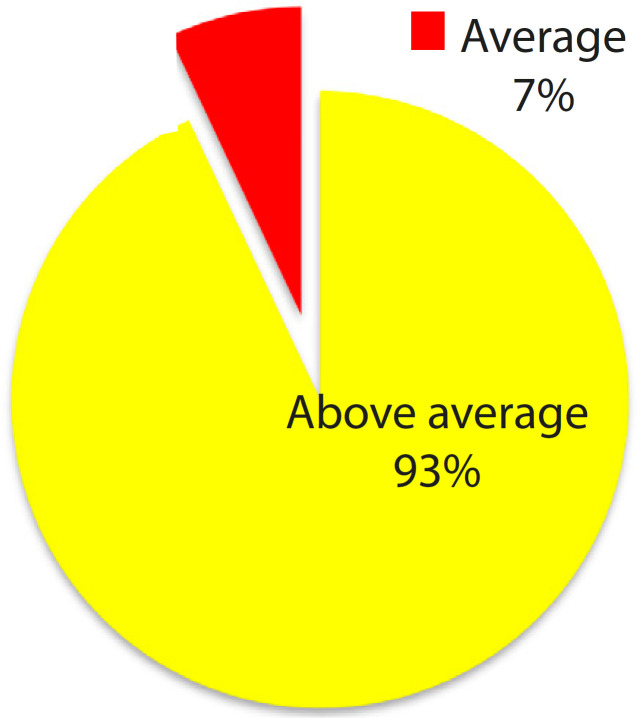
Scoring on accuracy of the model structures (n=30).

Figure 3.2 shows that 93% of the clinicians agreed that the accuracy of the heart model structures was above average (excellent and good). Structures such as the circumflex branch of left coronary artery, great cardiac vein, papillary muscle, and coronary sinus contributed to model accuracy of average. These results were influenced by adequate heart anatomical knowledge, especially the position of coronary sinus. The coronary sinus was only identified by 17 clinicians. Structures such as great cardiac vein and circumflex branch of left coronary artery run in the same cardiac groove; therefore, these structures were difficult to identify without colours.

Table 1 showed that 25 out of the 29 heart structures were identified correctly by all the 30 clinicians. Structures such as the circumflex branch of left coronary artery, great cardiac vein, papillary muscle, and coronary sinus each rated 77%, 70%, 70%, and 57% accurate.

**Table 1:** Identification of heart structures by clinicians (n=30).

Heart structures	Correct	Incorrect	Percentage (%)
Aortic valve	30	0	100
Arch of aorta	30	0	100
Ascending aorta	30	0	100
Brachiocephalic trunk	30	0	100
Chordae tendineae	30	0	100
Circumflex branch of left coronary artery	23	7	77
Coronary sinus	17	13	57
Descending aorta	30	0	100
Great cardiac vein	21	9	70
Inferior vena cava	30	0	100
Interventricular septum	30	0	100
Mitral valve	30	0	100
Left anterior descending artery	30	0	100
Left atrium	30	0	100
Left common carotid artery	30	0	100
Left pulmonary artery	30	0	100
Left pulmonary vein	30	0	100
Left subclavian artery	30	0	100
Left ventricle	30	0	100
Papillary muscle	21	9	70
Pulmonary valve	30	0	100
Right atrium	30	0	100
Right auricle	30	0	100
Right coronary artery	30	0	100
Right pulmonary vein	30	0	100
Right pulmonary artery	30	0	100
Right ventricle	30	0	100
Superior vena cave	30	0	100
Tricuspid valve	30	0	100

**Fig. 3.3:**
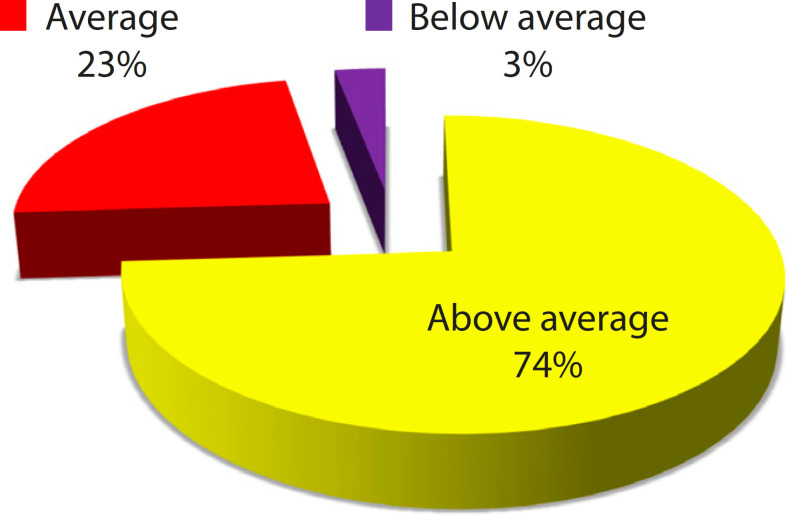
Scoring on heart model for treatment planning (n=30).

Figure 3.3 shows scoring on the heart model as a visual aid for planning of the treatment in cardiovascular disease. Seventy-four percent of the clinicians scored the model to be above average and only 3% scored it poor on usefulness of the model in treatment planning. Few clinicians suggested that future pathological cardiac model will be useful in the diagnosis and planning of operations, especially for congenital complex cardiac anomalies.

**Fig. 3.4:**
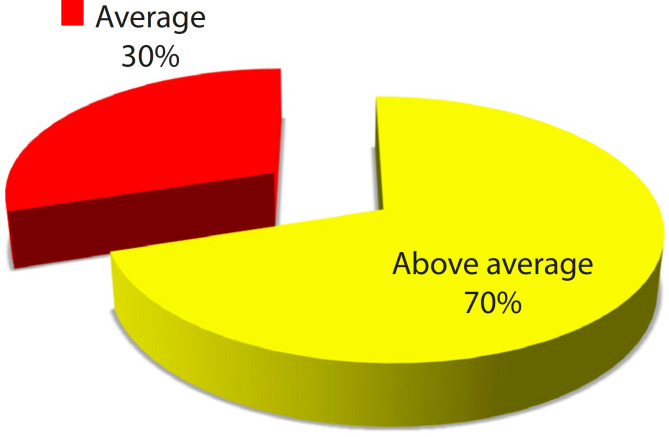
Scoring of heart model for training purpose (n=30).

One of the aims of creating a 3D prototype heart model is for training of junior doctors. 3D prototyping of the replicas certainly allows direct visual access to the physical structures and, more importantly, direct physical manipulation, such as practice surgery on the replica of the structure to be operated. Scoring of the model as ‘above average’ and ‘average’ for training purposes were 70% and 30%, respectively (Figure 3.4). Suggestion was given to change the model materials to more flexible transparent plastic, which can be cut easily during the training sections.

**Fig. 3.5:**
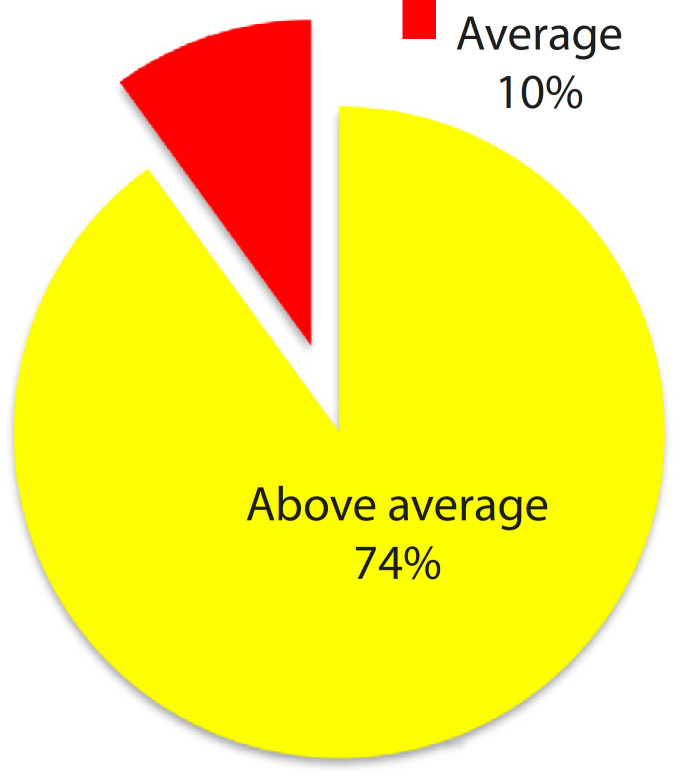
Scoring of heart model as a surgical visual aid.

The 3D prototype heart model allows easy understanding of both surgical and anatomical relations of the heart from the surgeon’s viewpoint. The physical models allow surgeons to predict difficulties and adverse events before operating. Figure 3.5 showed that the heart model scored 90% for above average as a surgical visual aid.

**Fig. 3.6:**
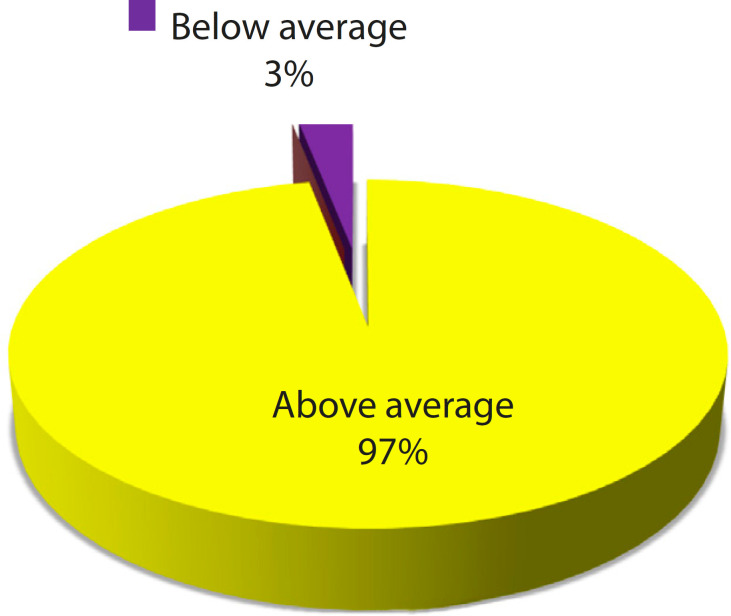
Scoring of the heart model as a teaching tool.

RPT are promising methods for improvement of anatomical education in medical students. Figure 3.6 shows that 29 clinicians (97%) rated this model above average for anatomical teaching tool.

**Fig. 3.7:**
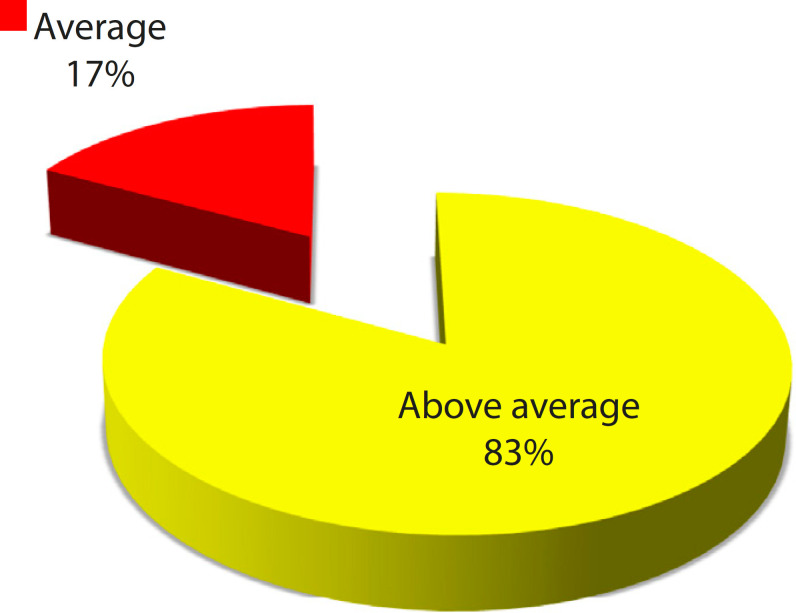
Overall score of the threedimensional heart model.

Overall, this 3D RP cardiac model was rated as excellent (33%), good (50%), and average (17%) (Figure 3.7).

## DISCUSSION

Generating 3D physical bio models from medical data is still an ongoing technological breakthrough. It has been applied in various fields of medicine and continues to generate new applications. Its usage on the human heart has just started a growing interest^[16]^.

Prior to surgery, in the preoperative stage, our 3D bio model is useful in diagnosing the condition of the patient. Traditional diagnosis of heart diseases are made using ECG, echocardiography, CT, and MRI scans; however, some procedures such as the CT angiogram are very challenging^[14]^, also, there is the presumed radiation side-effects from these scans. Using such 3D cardiac bio models, surgeons can now visualize the intricate structures before starting any interventional procedures. Being well prepared is a key factor to ensure surgical success and reduced morbidity.

Cardiac bio model provides great insights into the structural heart anomalies. These include hypertrophy and fibrosis, both of which are hallmark of cardiomyopathy. It has been particularly helpful with regard to complex anatomic structures and disorders that are not easily captured or understood in two dimensions^[16]^.

Many reports in the literature describe the use of models in the treatment of valve disease. Schievano et al.^[17]^ decide the patients’ suitability for percutaneous pulmonary valve implantation by assessing their 3D MRI-based models of their right ventricular outflow tract and pulmonary trunk.

Furthermore, by displaying the heart as a whole, this bio model allows overall view of the heart and helps in determining the overall severity of the disease state, especially in complex congenitally malformed hearts^[16,18]^. The bio model will not completely replace the previous methods, but it allows a greater confirmation on the diagnosis as it allows a physical view of the patient’s heart model.

Explaining heart pathology to the patients and other surgeons in the team is a crucial part of the communication process prior to surgery. This is to ensure all parties are aware and have full knowledge of the nature of the disease. Traditionally, this procedure is performed via explanation on the 2D images produced through CT scans between surgeons. Explanation to the patient on the other hand is carried out by doctors describing the patient using interpreted CT scans results or using generic teaching models. These current methods of communication pose several limitations. While the surgeons are able to understand the condition of the patient, however they have to rely on their imagination to plan and visualize the surgical process. As for the patients, their understanding of the nature of disease will be limited even after doctor’s verbal or descriptive explanation using CT scans, as they are unlikely to be knowledgeable in this particular field and probably have never seen an open heart surgery. This may result in underestimating the severity of the disease. In addition, it is likely that 2D images are ineffective in representing the complex 3D relationships in the actual heart disease^[19]^. Computer generated 3D models could resolve these issues, albeit it causes reliance on computer workstations and the flexibility of perceiving or trying to understand how the structure looks like. The 3D prototype cardiac bio model is able to overcome these limitations. By being physically examinable, the patients can precisely understand the obstacles that he or she might face, and the surgeons can directly use the bio model to provide exact information to the patients. Therefore this would bridge the communication barrier and minimize misunderstandings between doctor and patients^[20]^.

The clinical uses of RP are widespread. Because physical models allow both anatomic and clinical information to be conveyed in a visual and tactile form, the educational applications of RP remain limitless. The models are excellent teaching materials to all involved in cardiac imaging or surgery.

3D cardiac teaching models have long been used as method for anatomy teaching^[12,20]^. It gives a clear visual of how the heart is structured, albeit it lacks appropriate dimensions as the models were mostly of artist impression, and are produced through molds with a follow-up painting by artists^[20,21]^. Using cadaveric heart for teaching is also unfeasible due to cardiac collapse where the heart actually collapses and loses its actual structure after an individual deceases because of the pliant myocardium. Production of 3D prototypes of various pathologic conditions is even more important as there have been increasing restrictions to keep human body parts for teaching as well as clinical purposes and pathologic specimens are available only when they are removed at surgery or at autopsy.

This cardiac bio model is generated based on the CT scan, which is able to represent the structure of the myocardium accurately and is not based on artist’s impression. Thus, this bio model is more realistic and provides greater understanding of the structure. In addition, this model is based on the individual patient. This gives a clearer visual understanding of the concept of the scar-tissue disease as compared to verbal explanation. Moreover, 3D bio models of diseased hearts are not commonly found in the market; this makes it even harder for surgeons to explain to patients and relatives what is going to happen on the operating table.

Depending on their intended application (education, catheter navigation, device sizing, and testing), physical models may be printed in multiple materials using a variety of 3D printing technologies, each with its own specific feature^[22]^.

This model was created as similar as possible to a normal human heart. Therefore, the colours are not applied to identify the structures easily. For teaching purpose, the vessels can be coloured separately.

### Limitations and Future Work

This model was created only based on DICOM format of the CT scan slices. The state-of-the-art medical imaging facilities, such as MRI and echocardiography, provide precise digital information of the cardiovascular structures of the human body and need to be integrated to create future 3D prototype heart models. Sometimes it was quite difficult to decide where to draw the border between two tissues because of similar pixel colour. In such cases, the borders were drawn subjectively according to anatomy knowledge of the illustrator. This problem can be overcome by fusion of images from CT scans, MRI scans, and echocardiogram.

The anatomy of the cardiac valve leaflets was not appropriately integrated into the models because they are difficult to integrate using CT scan images. For certain anomalies, nonetheless, precise knowledge about the cardiac valve anatomy is invaluable. Future research must address this issue, and this might be better accomplished by fusing the images obtained by the tomographic techniques and images from the echocardiography.

Also, some problems arose in the small coronary vessels. The left and right coronary arteries, the circumflex artery, and the largest veins are visible, and one can follow such vessels from their origin to the periphery. On the periphery, coronary arteries and veins sometimes lie in close proximity and look like one large vessel. Some vessels did not have a uniform course in the 3D view. There were also some discontinuations, which occurred because they were not recognized on every cross-section and were omitted on some of them. There are some artifacts, such as spots painted on some cross-sections as vessels, but actually not corresponding to vessel tissues. They look like a single pixel or group of pixels apart from the coronary vessels.

These mistakes in the generated spatial heart model had to be corrected, either manually or automatically, by a computer program. It is very difficult to imagine the failures in space or to see their relationship to other tissues in space. It was expected that a kind of 3D editing would be of great help in improving the spatial heart model.

RP has also been used for the surgical and catheter-based treatment of pathologic abnormalities involving the aorta, including cases of complex abdominal aortic aneurysms. By creating a transparent aorta, endovascular procedures such as thoracic endovascular aortic repair can be used as a training model by the trainees.

Regarding the future, we are planning to create a normal and pathological combined heart-lung mediastinal model, which will be useful in preplanning management of lung or mediastinal mass. In addition to that, creating a heart model with enabling simulations of surgical procedures will be our focus.

## CONCLUSION

This 3D RP heart model will be a valuable source of anatomical education and cardiac interventional management. This model will improve diagnostic accuracy, assist in planning complex interventions, and aid in medical student and resident understanding of cardiac diseases.

RP represents the next evolution in advanced image processing and can potentially serve as an effective bridge between traditional medical imaging and actual patient anatomy.

**Table:** 

Authors' roles & responsibilities	
SK	Substantial contributions to the conception or design of the work; or the acquisition, analysis, or interpretation of data for the work; drafting the work or revising it critically for important intellectual content; agreement to be accountable for all aspects of the work in ensuring that questions related to the accuracy or integrity of any part of the work are appropriately investigated and resolved; final approval of the version to be published
RARM	Substantial contributions to the conception or design of the work; or the acquisition, analysis, or interpretation of data for the work; drafting the work or revising it critically for important intellectual content; agreement to be accountable for all aspects of the work in ensuring that questions related to the accuracy or integrity of any part of the work are appropriately investigated and resolved; final approval of the version to be published
RS	Substantial contributions to the conception or design of the work; or the acquisition, analysis, or interpretation of data for the work; drafting the work or revising it critically for important intellectual content; agreement to be accountable for all aspects of the work in ensuring that questions related to the accuracy or integrity of any part of the work are appropriately investigated and resolved; final approval of the version to be published
SS	Substantial contributions to the conception or design of the work; or the acquisition, analysis, or interpretation of data for the work; drafting the work or revising it critically for important intellectual content; agreement to be accountable for all aspects of the work in ensuring that questions related to the accuracy or integrity of any part of the work are appropriately investigated and resolved; final approval of the version to be published
YFAA	Substantial contributions to the conception or design of the work; or the acquisition, analysis, or interpretation of data for the work; drafting the work or revising it critically for important intellectual content; agreement to be accountable for all aspects of the work in ensuring that questions related to the accuracy or integrity of any part of the work are appropriately investigated and resolved; final approval of the version to be published
VM	Substantial contributions to the conception or design of the work; or the acquisition, analysis, or interpretation of data for the work; drafting the work or revising it critically for important intellectual content; agreement to be accountable for all aspects of the work in ensuring that questions related to the accuracy or integrity of any part of the work are appropriately investigated and resolved; final approval of the version to be published
